# Dermatofibrosarcoma Protuberans: An Updated Review of the Literature

**DOI:** 10.3390/cancers16183124

**Published:** 2024-09-11

**Authors:** Marcin Jozwik, Katarzyna Bednarczuk, Zofia Osierda

**Affiliations:** 1 Department of Gynecology and Obstetrics, Collegium Medicum, University of Warmia and Mazury in Olsztyn, 10-045 Olsztyn, Poland; 2 Scientific Circle of the Department of Gynecology and Obstetrics, University of Warmia and Mazury in Olsztyn, 10-045 Olsztyn, Poland; katarzyna.bednarczuk@student.uwm.edu.pl (K.B.); zofia.osierda@student.uwm.edu.pl (Z.O.)

**Keywords:** dermatofibrosarcoma protuberans, soft-tissue tumor, dermal sarcoma

## Abstract

**Simple Summary:**

Dermatofibrosarcoma protuberans (DFSP) is a rare proliferative condition representing skin sarcomas which is known to locally recur yet very rarely metastasizes. Its genetic background is a reciprocal translocation t(17;22)(q22;q13) that produces *COL1A1-PDGFB* gene fusion. Complete resection is the primary treatment. The aim of this reappraisal is to review various correlates of the condition. Our review underlines that cases of low-to-moderate grade DFSP with excellent prognosis must be distinguished from high grade fibrosarcomatous DFSP with a much worse prognosis.

**Abstract:**

Dermatofibrosarcoma protuberans (DFSP) is a rare proliferative condition representing skin sarcomas which is known to locally recur yet very rarely metastasizes. Its genetic background is a reciprocal translocation t(17;22)(q22;q13) that produces *COL1A1-PDGFB* gene fusion. Complete resection is the primary treatment. The aim of this review is to outline the pathogenesis, diagnosis, and management of DFSP. A clear-cut distinction between low-to-moderate-grade DFSP with excellent prognosis and high-grade fibrosarcomatous DFSP with a much worse prognosis is underlined. Malignant transformation within DFSP (or high histologic grade), older age, being female, large primary tumor size (≥10 cm), narrow surgical margins of excision (<3 cm), surgical margin positivity for tumor cells, short time to recurrence, numerous recurrences, tumor that was recently rapidly enlarging, and presence of pain in the tumor have all been proposed as clinicopathological risk factors for recurrence and metastasis. A tendency for local growth and local relapses of well- and moderately differentiated DFSPs is an argument for their surgical excision, possibly combined with reconstructive surgery, even in patients of advanced age. Another main point of this review is that cases of DFSP with fibrosarcomatous transformation are a challenge and require careful medical attention. Both anatomopathological evaluation of the presence of lymphovascular space invasion and sentinel lymph node biopsy at DFSP surgery merit further study.

## 1. Introduction

Dermatofibrosarcoma protuberans (DFSP) is an uncommon skin and/or soft-tissue malignancy. When borne in the skin, it usually stems from the dermis and subcutaneous tissue, but not from the epidermis. As a soft-tissue tumor, it may involve subcutaneous fat or connective tissue [1,2]. Eisen and Tallini state that DFSP originates in the reticular dermis [3]. Fibroblasts or histiocytes are believed to be the cells of its origin, yet the neoplasm has frequently been referred to as a fibrohistiocytic tumor without defining its precise histogenesis [3,4,5,6,7,8,9]. Other likely precursors are perineural cells [10,11]. It was first recognized as a separate entity independently by Sherwell and Taylor in 1890 and later described in much detail by Darier and Ferrand in 1924; nonetheless, the full term ‘dermatofibrosarcoma protuberans’ was proposed by Hoffmann in 1925 [12,13,14,15]. The 2012 guideline by the National Comprehensive Cancer Network (NCCN) states that ‘typical’ DFSP is an uncommon, low- to intermediate-grade sarcoma of fibroblast origin [16].

Information on the incidence patterns of DFSP is still somewhat limited. In a series of 240 patients from the Memorial Sloan-Kettering Cancer Center, New York, NY, there was equal distribution between men and women [17]. Population-based data from the Surveillance, Epidemiology, and End Results (SEER) Program for 1973 to 2002 showed that DFSP’s overall annual incidence in the United States was 4.2 per million inhabitants. Women had slightly higher rates of incidence than men (4.4 vs. 4.2 per million per year), the difference being of borderline statistical significance. Interestingly, an ethnic disparity was detected, with annual incidence among African Americans (6.5 per million) being almost double the incidence among Caucasians (3.9 per million) [18]. Data from other SEER Program registries reviewed for the period from 1992 to 2004 demonstrated an overall DFSP incidence of 4.5 per million inhabitants per year and confirmed a higher incidence in women than in men (4.7 vs. 4.4 per million per year, respectively) and in African Americans than in other races [19]. The tumor can occur at any age, yet its incidence peak was observed in patients in their 40s [19]. The largest population-based study of DFSP so far, derived from a cohort of almost 7000 patients observed in the years 2000 to 2010, confirmed its higher incidence among women and African Americans [20]. Somewhat similar trends were shown by data from the Alberta Cancer Registry, Alberta, Canada, where the overall incidence was roughly twice as high as previously reported, 9.3 per million per year. Over the study period (1988 to 2007), this incidence was found to increase by 3.2% per year in women and decrease by 2.7% per year in men [21]. The available European data are as follows: over 3 cases per million inhabitants per year in Eastern France, with men affected 1.2 times more often than women [22], and a more frequent incidence in men than in women (4.4 vs. 4.0 per million per year, respectively) in Sweden [23]. The annual incidence in Denmark for the years 2000–2012 was 5.3 cases per million inhabitants [24]. This relative rarity of DFSP is reflected in the lack of prospective scientific evidence.

Yet, even if infrequent, DFSP represents the most common dermal sarcoma (about 1% of all soft-tissue sarcomas), more than 1% of all head and neck malignant tumors, and 7% of all head and neck sarcomas [25,26].

## 2. Review of the Literature

### 2.1. Clinical Presentation

The clinical picture of DFSP varies from plaquelike or small nodular lesions to large uneven masses [27]. Usually, it is a slow-growing, firm, multinodular lesion. The description ‘protuberans’ in the name of the entity refers to lumps of tissue that form under the surface of the skin [28]. Yet, it has been observed that some cases of DFSP do not protrude above the skin. Consequently, the French Group for Cutaneous Oncology described several variants of the preprotuberant stage of the lesion. These were varied nonprotuberant plaques lasting for a mean of 7 years before the neoplasm became apparently bulging and nodular [29]. We view these preprotuberant variants as precancers.

Typical or low-to-moderate-grade DFSP is generally characterized by slow growth with a tendency to infiltrate surrounding tissues and to persistently recur locally over time, whereas regional and distant metastases are rare. The tumor cells invade subcutaneous tissue in the form of tentacle-like, or villous, projections through the skin septa and fat lobules and surround the skin appendages. These tumor extensions contain few cells and initially look similar to normal fibrous tracts, which makes difficult to determine the true extent of the lesion [3,30]. This infiltration in subcutaneous fat often resembles a honeycomb pattern [31,32,33]. The longest preoperative duration of DFSP reported in the literature was 60 years [34]; the longest interval from primary excision to local recurrence was 16 years [35]. The pattern of tumor site location is presented in Table 1.

A definitive diagnosis is first made by incisional (true-cut) or, less frequently, excisional biopsy in combination with anatomopathological examination, including histologic morphology and immunohistochemistry staining. Fine-needle aspiration biopsies are less frequently performed nowadays, possibly due to the relative inaccuracy of cytologic smears to render the DFSP diagnosis out of a plethora of other spindle cell lesions occurring in the skin and soft tissue [37]. Histologically, DFSP is similar to benign fibrous histiocytoma and deeply infiltrating dermatofibroma. Giant-cell fibroblastoma, occurring predominantly in the first decade of life, is considered a juvenile form of DFSP by some, yet not all, authors [38,39]. Llombart et al. believe that many cases diagnosed in adults actually begin in childhood [30].

### 2.2. Pathological Findings

Under a microscope, DFSP tumor cells present as uniform spindle cells arranged in a characteristic well-defined storiform or cartwheel-like pattern; they have minimal cytologic atypia, uniform nuclei, absent-to-low mitotic activity, and characteristically stain intensely for CD34 [1,3,36,40,41,42,43]. CD34 is a monomeric 115 kDa glycoprotein primarily expressed on normal hematopoietic progenitor cells. A further immunohistochemical profile of DFSP cells includes positive staining for apolipoprotein D and nestin and negative staining for factor XIIIa, stromelysin III, HMGA1, HMGA2, tenascin, D2-40, and CD163 [30]. Possible histologic variants of the neoplasm include pigmented (or Bednář tumor), myxoid, myoid, granular = cell, sclerotic, and atrophic DFSP, giant-cell fibroblastoma, and DFSP with fibrosarcomatous transformation [30,44,45]. However, some authors argue that sclerosis in DFSP may represent an expression of spontaneous tumor regression and discredit the existence of the sclerotic variant of the neoplasm [46]. Composite tumors have also been described [8,47].

Importantly, the above microscopic picture differs in case of fibrosarcomatous transformation. Then, fibrosarcomatous foci look as follows: the cells are mostly fusiform with enlarged atypical nuclei and are often CD34 negative, and cytologic atypia and a high mitotic count are present. In contrast to the storiform pattern in classical DFSP, fibrosarcoma cells grow in a herring-bone arrangement [6,7,34,48]. After Enzinger and Weiss, an important criterion of the diagnosis is that the fibrosarcomatous component must be present in at least 5% of the tumor’s volume, whether primary or recurrent [16,34,49,50]. Reports by pathologists indicate that such lesions can be with partial fibrosarcomatous transformation or purely fibrosarcomatous DFSPs encompassing the whole tumor mass [34,45,50]. In the Abbott study of 41 cases, fibrosarcomas arose in 38 (93%) de novo tumors and in 3 (7%) recurrent tumors [50]. In the fibrosarcomatous component of 18 DFSPs from the Goldblum study, 17 resembled fibrosarcoma and 1 malignant fibrous histiocytoma. All these tumors expressed CD34 in the DFSP component but only nine (50%) in the fibrosarcomatous component [42]. In an earlier smaller series, mere focal CD34 immunoreactivity was present in the fibrosarcomatous portion in only one (14%) of seven tumors [41]. In the Mentzel study, only 15 (45%) of 33 fibrosarcomatous lesions demonstrated CD34 positivity [34]. This loss of CD34 expression of varied degree is in line with dedifferentiation in high-grade tumors. Of note, fibrosarcomatous transformation has been linked with the overexpression of p53 protein [6,45,50]. One study suggested the loss of the honeycomb pattern in subcutaneous fat infiltration during fibrosarcomatous change [31]. In another study, necrosis, a high mitotic rate (>10 mitoses per 10 high-power fields), and the presence of pleomorphic areas in fibrosarcomatous DFSPs tended to be related to poor clinical outcomes [34]. Therefore, whether necrosis is a distinctive feature of high-grade DFSP requires confirmation. Table 2 outlines the principal differences between typical DFSPs and cases with fibrosarcomatous transformation. Clearly, fibrosarcomatous transformation heralds a potential for a more aggressive course.

Angouridakis et al. stated that approximately 85–90% of all DFSPs are low-grade tumors, whereas the remaining 10–15% contain a high-grade tumor component. This transformation, when presenting in more than 5% of the tumor’s volume, leads to a higher incidence of local relapse and distant metastasis [25]. Consequently, the unusual clinical behavior of transformed DFSPs has received special attention from researchers. The most frequent seems to be the above-mentioned fibrosarcomatous change, yet it is not the only one possible. For example, in a Hungarian study of eight lesions with de novo sarcomatous change present, there were five fibrosarcomatous and three malignant fibrous histiocytomatous transformations [51]. Another report described a typical DFSP with several slowly growing recurrences progressing to malignant fibrous histiocytoma over a period of 13 years [4]. Nowadays, malignant fibrous histiocytoma is frequently referred to as pleomorphic dermal sarcoma.

The clinical behavior of the transformed tumor is difficult to predict. Rare studies where histologic comparisons of primary lesions with their consecutive local recurrencies were conducted strongly suggest a gradual replacement of the typical storiform pattern by the fibrosarcomatous herring-bone pattern [52]. Dedifferentiation (that is, the loss of initial differentiation) and increased nuclear atypia and mitoses have been observed in recurrent and metastatic tumors [52].

### 2.3. Genetic Studies

Of interest, genetic background can be detected in the vast majority of DFSP cases. Early research established that more than 90% of all DFSPs arise from unique unbalanced reciprocal translocations of chromosomes 17 and 22: t(17;22)(q22;q13) and usually demonstrate a supernumerary ring chromosome or chromosomes derived from t(17;22). This genetic alteration can be detected by karyotype, fluorescence in situ hybridization (FISH), or reverse-transcription polymerase chain reaction. The FISH technique is superior and recommended as a routine diagnostic tool, especially in cases with unusual histopathological subtypes and/or immunohistochemical features [53]. The above chromosomal rearrangement results in the chimeric fusion between the collagen type Iα1 gene (*COL1A1*) and the platelet-derived growth factor β-chain gene (*PDGFB*) (specifically, in the fusion of exon 2 of *PDGFB* to various exons of *COL1A1*) [54,55,56]. The gene product, a COL1A1-PDGFβ fusion protein, binds to the constitutively expressed PDGF receptor and causes a constant activation of PDGF receptor β protein tyrosine kinase, which in turn promotes cell growth in DFSP via autocrine growth stimulation [57,58,59]. Paracrine growth stimulation is also taken into account [30,60]. Further, in an interesting investigation from Sweden, gene expression profiling of tumors with *COL1A1*-*PDGFB* fusions identified an array of transcriptionally up-regulated genes in the amplified regions of chromosomes 17 and 22 [61]. Studies using a combination of RNA sequencing and FISH indicated that, apart from the canonical *COL1A1*-*PDGFB* fusion, a collagen type VI α-3 (*COL6A3*)-platelet-derived growth factor D (*PDGFD*) gene fusion is possible and seems to predispose to DFSP localization in the breast [62]. Moreover, a large-scale Taiwanese study on 188 cases identified additional examples of cryptic *PDGFB*-rearranged and alternative *PDGFD*-rearranged DFSPs, including *COL6A3*-*PDGFD*-positive cases (again, showing a preference for the female trunk, including the breast) and Elastin Microfibril Interfacer 2 (*EMILIN2*)-*PDGFD*-positive cases (which are male-predominant and mostly fibrosarcomatous) [63]. A recent Chinese work detected a heterozygous germline mutation of *ERCC2* in a 19-year-old man with DFSP [64]; *ERCC2* is a protein involved in transcription-coupled nucleotide excision repair.

### 2.4. Metastatic Potential and Recurrence

Metastatic DFSP is rare, believed by some to occur in no more than 6% of all cases [65,66]. The rarity of metastatic disease has been linked to DFSP’s superficial location, low-to-intermediate histologic grade in the vast majority of cases, and generally small size [65]. Metastasis primarily occurs via the hematogenous route. It typically spreads to the lungs [66,67], yet bones, including the rib cage, the brain, and the heart are other known sites of spread [66]. The detailed postmortem studies published are the most saddening because they testify that such a systemic spread can be virtually to all organs and tissues [3,52,68]. From one study, unusual sites of metastasis were the peritoneum, axillary lymph node, and thoracic vertebrae [49]. The course of the generalized disease can be fulminant [52].

Rare instances of lymphatic spread have been reported as singular case reports. For example, in 1974, Bonnabeau et al. presented a DFSP patient who developed multiple pulmonary and pleural metastases [69]. In a 1975 review of the world literature, Brenner et al. found 17 patients with hematogenous spread and six patients with spread to the lymph nodes, and these authors reported a seventh patient with regional node metastases at that time [70]. Three years later, Kahn et al. published an update of the account, stating as follows: ‘More than 400 patients with dermatofibrosarcoma protuberans have been reported in the literature, including the present case, five of these patients had lymph node metastases, 17 patients had hematogenous spread, and three had both lymphatic and blood-borne metastases’ [71]. Although infrequent, both hematogenous and lymphatic metastases emphasize the need for a long-term follow-up of the DFSP patient after surgical excision. Unfortunately, distant metastases can arise after adequate surgery and without prior local recurrence of the primary tumor [3,71].

A systematic review comparing oncologic outcomes for DFSP with and without fibrosarcomatous transformation confirmed that the risk of local recurrence, metastasis, and death is significantly elevated in fibrosarcomatous DFSP as compared with DFSP. These are the actual patients who may die from the disease. Therefore, the fibrosarcomatous change in DFSP warrants aggressive treatment and close observation for recurrence and metastasis [48]. Sentinel lymph node biopsy has been advocated in cases with fibrosarcomatous transformation [25]. Unfortunately, this transformation is often not recognized until the final histologic diagnosis [26].

A large retrospective cohort study on 188 patients established that recurrence-free survival is highly significantly shorter in tumors with fibrosarcomatous transformation than in typical tumors. Metastatic disease was seen in no patients with typical lesions, whereas it was confirmed in 17.6% (3 of 17) of patients with fibrosarcomatous transformation (*p* < 0.001) [72]. Other exceptions with a high metastatic rate were the McPeak study, where 5 (5.8%) of 86 patients developed metastases and all of them died of the disease [27], and the Abbott study, where 4 (9.8%) of 41 patients developed metastases and 2 patients died of the disease [50]; however, these were selected populations. A retrospective study on 368 patients from two tertiary sarcoma centers found that, in comparison with patients with typical DFSP, patients with fibrosarcomatous transformation were older by approximately 8 years, female, and with larger, painful tumors that were recently rapidly enlarging [26].

A large analysis of 5249 DFSP cases from the National Cancer Database, USA, for the years 2003–2012 looked not only at clinicopathologic but also socioeconomic corelates. In brief, 3.1% of patients died during an average of 51 months of follow-up, yet this figure did not represent cancer-specific mortality only. Mortality from DFSP was significantly associated with lack of insurance, Medicaid and Medicare insurance, high-grade tumor histology, and positive postoperative margins. Interestingly, specialist treatment at an Integrated Network Cancer Program predicted survival. Higher odds of postoperative radiation therapy were associated with large tumor size, high-grade histology, and positive postoperative margins and inversely associated with treatment at high-volume facilities and non-head and neck tumors. Higher second surgery rates were associated with Hispanic ethnicity, and lower rates were associated with the female sex [73].

In a report combining patient data from three sarcoma treatment centers—the Royal Marsden Hospital, London, UK; the Netherlands Cancer Institute Antoni van Leeuwenhoek, Amsterdam, The Netherlands; and the Erasmus Medical Center-Cancer Institute, Rotterdam, The Netherlands—357 DFSP patients were identified, of whom 41 (11.4%) had a histopathologically confirmed fibrosarcomatous transformation, 4 (1.1%) developed distant metastases at a median time of 68 months, and 2 (0.6%) died of the disease. Patients with transformed lesions had significantly larger tumors and more often received radiotherapy as a treatment modality [67].

In the study by Hayakawa et al., a large size of the primary tumor was found to be a statistically significant risk factor for metastases in DFSP. Moreover, these authors reported on a 37-year-old patient who developed lung metastases from a 5 cm tumor with a histology of typical DFSP without fibrosarcomatous transformation and eventually died of the disease. Consequently, in their series of 67 patients, the metastasis rate was calculated to be 1.7% for typical DFSPs and as high as 57% for fibrosarcomatous cases [49]. We are cautious to accept that typical DFSPs can metastasize. We believe that fibrosarcomatous transformation into the least differentiated high histologic grade (G3) is necessary for the generalized spread of malignant cells. Therefore, we speculate that the sole patient from the Hayakawa study who developed metastases from a typical DFSP could have had a tumor which contained the fibrosarcomatous component at slightly less than 5% of the tumor’s volume. Then, anatomopathologic diagnosis with the current criteria would still be typical DFSP, even with some fibrosarcomatous foci present.

Systemic dissemination is strongly associated with previous tumor recurrence and fibrosarcomatous transformation within DFSP [57]. In their review of 115 cases, Taylor and Helwig noted that lymphovascular space invasion is rare in DFSP [36]. Let us underline that lymphovascular space invasion can be focal or substantial and will occur in cases of metastatic potential only, that is, in DFSPs with fibrosarcomatous transformation. Therefore, material from cases with fibrosarcomatous transformation should be examined for the presence of substantial lymphovascular space invasion as a risk factor for and in the early event of generalized disease.

### 2.5. Differential Diagnosis

Initial errant diagnoses of DFSP may include a wide spectrum of entities: cellular dermatofibroma, cellular leiomyoma, cellular neurofibroma, neurofibroma, fibrosarcoma, low-grade sarcoma, low-grade leiomyosarcoma, low-grade malignant schwannoma, low-grade malignant peripheral nerve tumor, desmoplastic melanoma, spindle cell tumor, and dedifferentiated liposarcoma [1,45]. Llombart et al. added protruding scar, morphea, morpheaform basal cell carcinoma, atrophoderma, and vascular malformations to this list [30]. The cytologic differential diagnosis of DFSP was presented by Domanski and Gustafson [37]. Initial misdiagnosis, prolonged time to accurate diagnosis, and large tumor size at diagnosis are common [16].

### 2.6. Staging and Grading

To date, there is no definitive staging system for DFSP. Some authors apply the American Joint Committee on Cancer (AJCC) Staging Protocol for Sarcoma of Soft Tissue classification [49]. Others studies use a straightforward distinction: primary tumor vs. local recurrence [17]. The NCCN classification is as follows: stage I is local disease, stage II is regional disease, and stage III is distant disease [16]. Somewhat similarly, the 2018 German dermatologic–oncologic practice guidelines provide the following simplified classification: stage I—primary tumor (localized disease); stage II—lymph node metastasis; stage III—distant metastasis [74].

There is a role for imaging techniques in DFSP. Both ultrasound (7.5–10 MHz) and magnetic resonance imaging can be useful in assessing the extent of the tumor prior to surgery. With suspected local recurrent and/or distant disease, lymph node ultrasound and cross-sectional imaging by computed tomography or magnetic resonance are of value [74].

### 2.7. Treatment

Complete resection is the primary treatment. The modern options include wide local excision (WLE), Mohs micrographic surgery (MMS), and surgery followed by three-dimensional complete circumferential and peripheral deep margin assessment (or CCPDMA) [66,75,76]. All these techniques rely on the microscopic evaluation of the excised surgical margins, which are possibly narrowest for CCPDMA, medium for MMS, and widest for WLE. After CCPDMA, a Tubingen torte technique on slices from paraffin-embedded tissue taken from the entire circumferential and deep surgical margins with a special staining offers a refinement of final histopathologic assessment. Another variant of Mohs surgery, a slow Mohs surgery (SMS), involves excision of the tumor tissue layer by layer until all the tumor cells are removed, and it has proven to be effective in skin cancer. Some studies note that in some patients with vulvar DFSP, a radical vulvectomy was performed [1,65,77]. A systematic review gave a weak recommendation in favor of MMS over WLE for DFSP excision [76]. In MMS, the excised fresh specimen is frozen and sliced into serial sections for microscopic evaluation of complete margins. There is accumulating evidence that modified MMS with horizontal en face sectioning of formalin-fixed paraffin-embedded blocks, known as slow Mohs surgery, provides better results in terms of complete resection and tissue-sparing capabilities [78,79,80]. Yet the use of MMS is not indicated for patients with fibrosarcomatous transformation [26,72]. In the Ratner study, as many as 80% of patients required several staged Mohs excisions to achieve complete tumor clearance [81].

An important piece of information came from a recent systematic review with a meta-analysis of eight eligible cohort and case–control studies: the performance of ≥3 cm margins in decreasing the local relapse rate was found to be highly significantly better (*p* < 0.00001) than that of <3 cm margins [58]. This is in keeping with a detailed microscopic study with three-dimensional modeling. The likelihood that a given width of excision around the macroscopic DFSP tumor would clear its entire microscopic extent was determined to be as follows: a width of 1 cm around the primary tumor would have left microscopic residual mass in 70.7% of cases; a width of 2 cm in 39.7%; 3 cm, 15.5%; and 5 cm, 5.2% [81]. Goldblum et al. put forth an interesting hypothesis that tumors in which clear margins are achieved may represent a less aggressive subset of DFSPs [42].

Accordingly, there is little doubt that local spread and recurrence are due to inadequate excision, namely, failure to perform complete excision [25,82,83,84]. Three-dimensional reconstruction of DFSPs has demonstrated tricky tumors with highly irregular shapes and frequent finger-like, or tentacle-like, extensions [81]. These characteristic villous patterns of extension into the subcutaneous fat, fascia, and muscles, and, at the same time, the intention of the surgeon to preserve healthy tissue from resection, represent a surgical challenge [25]. Pathologists indicate that, because of the protuberant, infiltrative growth and low-grade cytologic features of typical DFSP, accurate microscopic assessment of margins can be challenging [45].

The understanding of the role of PDGF receptor β protein tyrosine kinase in DFSP has opened up new possibilities for tailored neoadjuvant therapies. Accordingly, since 2006, imatinib mesylate (Gleevec) has been approved by the Food and Drug Administration in the United States and later by European authorities as a single agent for the treatment of inoperable, primary, locally inoperable, recurrent, and metastatic DFSP [57,66,74]. Imatinib is a tyrosine kinase-inhibitor specifically directed at receptors for Colony Stimulating Factor 1 and PDGFR α and β [60]. The initial results of a pooled analysis of two phase II clinical trials were that the agent induced an objective response rate in approximately 50% of the cases [85]. A later retrospective analysis from a leading Polish oncologic institution on the vicissitudes of 15 patients with advanced DFSP treated with imatinib demonstrated the following results: ten (67%) partial responses, two (13%) stable diseases, and three (20%) progressive diseases [60]. In some patients with advanced disease, imatinib was effective in decreasing the tumor size and making the lesion operable. Seven (47%) out of these fifteen patients underwent resection of residual disease and remained free of disease [60]. In another series, thirteen (47%) patients underwent resection of residual disease following imatinib treatment and nine of them remained free of disease, even though the medication was discontinued [86]. A systematic review on imatinib for locally advanced or metastatic DFSP found that of 152 patients, complete response was observed in 8 (5.2%), partial response in 84 (55.2%), stable disease in 42 (27.6%), and progression in 14 (9.2%). A daily dose of 400 mg imatinib seems more appropriate than that of 800 mg; this is in relation to the adverse and severe adverse effects present in the vast majority of patients [87]. One conclusion of the review was that the medication is useful in inoperable DFSP patients [87]. In a French study from 2021, 27 patients with locally advanced DFSP received the tyrosine kinase inhibitors imatinib or pazopanib for a median duration of 7 months; 24 (89%) of them then underwent surgical excision, and during an over 5-year median follow-up, 23 (85%) patients were disease-free [88].

Because DFSP is relatively sensitive to radiation, radiation therapy may be an important treatment option for inoperable tumors or when postoperative margins are microscopically or macroscopically positive, or for tumors with multiple recurrences. Such a therapy may also be considered a primary treatment option for patients in whom wide excision would result in significant cosmetic or functional impairment. The target volume includes the tumor itself plus any scar(s) from previous surgery plus safety margins of 3–5 cm. With curative intent, a single dose of 2 Gy is usually administered five times a week for a total dose of 60–66 Gy (when microscopic evidence of tumor cells exists) or 66–70 Gy (when there is macroscopic evidence of residual tumor). In a palliative setting, and depending on tumor site and surrounding vital structures, a total dose of 50 Gy may be sufficient [74]. Adjuvant radiation of the tumor bed following the excision of DFSP is not recommended. A systematic review of 12 retrospective clinical trials showed only a weak trend towards lower local recurrence rates after adjuvant postoperative radiotherapy compared to surgery alone [89].

### 2.8. Prognosis

Let us underline that the existing hard data for the overall prognosis in DFSP are reassuring. Many reported DFSP cohorts noted no presence of metastases nor demise from disease. In an Italian report on 218 patients, the crude cumulative incidence of sarcoma-specific mortality at 5 and 10 years was 1.5% and 2.8%, respectively [83]. The relative 5-year survival in American population-based studies was excellent, at 99.2% (95% confidence intervals: 98.3–100%) [18]. In the Kreicher study, the 10-year relative survival of DFSP patients was 99.1% (95% confidence intervals: 97.6–99.7%) [20]. This generally favorable survival prognosis was confirmed in a recent retrospective analysis where information from the SEER Program for the years 2000–2018 was used. In a cohort of 7567 patients, 89 (1.18%) died of the disease. Significantly higher DFSP-specific mortality was associated with ≥10 cm tumor size and high histologic grade (7.07% and 10.08%, respectively) [2].

European guidelines recommend follow-up examinations every 6 months for the first 5 years and at yearly intervals thereafter for up to 10 years. In addition, imaging is reserved for recurrent DFSP or DFSP with fibrosarcomatous transformation, yet without clarification on the interval or type of imaging [57]. The NCCN Guidelines recommend follow-up visits every 6 to 12 months, with regular imaging for patients with high-risk features. Unfortunately, these guidelines do not specify what these high-risk features are, nor do they describe the length of follow-up [12]. In a Dutch study, routine chest X-ray during follow-up did not seem to be useful in patients with typical DFSP, except for those who had fibrosarcomatous changes and thus a relatively high risk for lung metastases [67]. Any suspicious region requires rebiopsy [16]. Patient education about regular self-exams is advised [16].

## 3. Conclusions

In summary, DFSP is a proliferative condition representing skin sarcomas which are known to locally recur yet very rarely metastasize. It is observed with an incidence of several cases per million inhabitants per year. The vast majority of DFSPs (~90%) harbor unbalanced reciprocal translocations of chromosomes 17 and 22: t(17;22)(q22;q13). Usually, it is a slow-growing, firm, multinodular lesion. Unfortunately, a typical low-or-moderate histologic grade DFSP can undergo sarcomatous transformation to fibrosarcoma or malignant fibrous histiocytoma and such a variant differs both in its histologic composition and clinical behavior. Then, its metastatic spread to all possible organs can be hematogenous and lymphatic and can be the cause of death. Malignant transformation within DFSP (or high histologic grade), older age, being female, large primary tumor size (≥10 cm), narrow surgical margins of excision (<3 cm), surgical margin positivity for tumor cells, short time to recurrence, numerous recurrencies, a tumor that was recently rapidly enlarging, and presence of pain in the tumor have all been proposed as clinicopathologic risk factors for recurrence and metastasis. Wide radial surgical excision with at least 3 cm margins is the gold-standard curative treatment for localized DFSP but may result in functional impairment or cosmetic disfigurement. When needed, skin flap transplantations should be considered, regardless of the patient’s age.

## 4. Future Directions

A repeated surgical excision of low-to-moderate-grade DFSP may offer good local control but may require skin reconstruction with flaps to cover a defect. Such a transplantation should be considered regardless of the patient’s age. Cases of typical DFSP with excellent prognosis must be distinguished from fibrosarcomatous DFSP with a much worse prognosis. Adequate staging and grading may be enhanced with modern approaches: anatomopathological evaluation of the presence of lymphovascular space invasion and sentinel lymph node biopsy at DFSP surgery. Both techniques merit further study in fibrosarcomatous DFSP. Possibly, tyrosine kinase inhibitors other than imatinib will offer better disease control in advanced DFSP.

## Figures and Tables

**Table 1:** Pattern of DFSP tumor site locations in the body.

Number of Patients	Upper Extremity	Lower Extremity	Trunk	Head or Neck	Other Location
*n* = 240 [17]	29%	23%	31%	14%	2%
*n* = 368 [26]	34%	29%	37%	-	-
*n* = 115 [36]	25%	26%	41%	15%	-

**Table 2:** Principal clinicopathologic differences between typical dermatofibrosarcoma protuberans (DFSP) and DFSP with fibrosarcomatous change. G1—well differentiated, G2—moderately differentiated, G3—poorly differentiated.

Feature	Typical DFSP	DFSP with Fibrosarcomatous Change
Histologic grade	Low to moderate (G1–G2)	High (G3 present in at least 5% of the mass)
Mitotic activity	Absent to low	High
CD34 positivity	Always present	Often absent
p53 expression	Low-level	Increased
Cellular arrangement	Storiform pattern	Herring-bone pattern
Tumor behavior	Indolent growth of a small mass	Often quick growth of a larger mass
Presence of pain	Rarely present	Often present
Interval to recurrence	Longer	Shorter
Distant metastases	Never or very rarely present	Can be present
